# Electrophysiological evaluation of the neuromuscular junction: a brief review

**DOI:** 10.1055/s-0043-1777749

**Published:** 2023-12-29

**Authors:** João Aris Kouyoumdjian, Eduardo de Paula Estephan

**Affiliations:** 1 Faculdade de Medicina de São José do Rio Preto, Departamento de Ciências Neurológicas, Psiquiatria e Psicologia Médica, São José do Rio Preto SP, Brazil.; 2 Universidade de São Paulo, Departamento de Neurologia, São Paulo SP, Brazil.; 3 Faculdade de Medicina Santa Marcelina, São Paulo SP, Brazil.

**Keywords:** Neuromuscular Junction, Myasthenia Gravis, Repetitive Nerve Stimulation, Single-Fiber Electromyography, Jitter, Junção Neuromuscular, Miastenia Gravis, Estimulação Nervosa Repetitiva, Eletromiografia de Fibra Única, Jitter

## Abstract

The nerve terminal and muscle membrane compose the neuromuscular junction. After opening the voltage-gated calcium channels, action potentials from the motor axons provoke a cascade for the acetylcholine release from synaptic vesicles to the synaptic cleft, where it binds to its receptor at the muscle membrane for depolarization. Low amplitude compound muscle action potential typically presents in presynaptic disorders, increasing by more than 100% after a 10-second effort in the Lambert-Eaton myasthenic syndrome and less in botulism. Needle electromyography may show myopathic motor unit action potentials and morphological instability (“ *jiggle* ”) due to impulse blocking. Low-frequency repetitive nerve stimulation (RNS) is helpful in postsynaptic disorders, such as myasthenia gravis and most congenital myasthenic syndromes, where the number of functioning acetylcholine receptors is reduced. Low-frequency RNS with a decrement >10% is abnormal when comparing the 4th to the first compound muscle action potential amplitude. High-frequency RNS is helpful in presynaptic disorders like Lambert-Eaton myasthenic syndrome, botulism, and some rare congenital myasthenic syndromes. The high-frequency RNS releases more calcium, increasing the acetylcholine with a compound muscle action potential increment. Concentric needle records *apparent* single-fiber action potentials (spikes). A voluntary activation measures the jitter between spikes from two endplates. An electrical activation measures the jitter of one spike (one endplate). The jitter is the most sensitive test for detecting a neuromuscular junction dysfunction. Most neuromuscular junction disorders are responsive to treatment.

## Resumo

O nervo terminal e a membrana muscular compõem a junção neuromuscular. Após a abertura dos canais de cálcio dependentes de voltagem, os potenciais de ação do axônio motor provocam uma cascata de eventos que libera acetilcolina das vesículas para a fenda sináptica, ligando-se ao receptor na membrana muscular para despolarização. O potencial de ação muscular composto de baixa amplitude ocorre nas desordens pré-sinápticas, aumentando em mais de 100% após esforço de 10 segundos na síndrome miastênica de Lambert-Eaton e menos no botulismo. A eletromiografia pode mostrar potenciais de ação da unidade motora miopáticos e instabilidade morfológica (“ *jiggle* ”) devido ao bloqueio do impulso. Estimulação nervosa repetitiva (ENR) de baixa frequência é útil nos distúrbios pós-sinápticos, como miastenia gravis e a maioria das síndromes miastênicas congênitas, quando há número reduzido de receptores de acetilcolina funcionantes. ENR de baixa frequência com decremento >10% é anormal comparando-se à amplitude do quarto com o primeiro potencial de ação muscular composto. ENR de alta frequência é útil nas doenças pré-sinápticas, como síndrome miastênica de Lambert-Eaton, botulismo e algumas síndromes miastênicas congênitas raras. ENR de alta frequência libera mais cálcio, aumenta acetilcolina, resultando em incremento do potencial de ação muscular composto. O eletrodo de agulha concêntrico registra potenciais de ação *aparente* de fibra única (PAAFU). Ativação voluntária mede jitter entre dois PAAFUs (duas junções neuromusculares). Ativação elétrica mede jitter de um PAAFU (uma junção neuromuscular). Jitter é o teste mais sensível para detectar disfunção de junção neuromuscular. A maioria dos distúrbios juncionais é responsiva ao tratamento.

## INTRODUCTION

Neuromuscular junction (NMJ) disorders have a common reduced safety factor (SF). Two ancillary electrophysiological tests can evaluate the NMJ: repetitive nerve stimulation (RNS) and single-fiber electromyography (SFEMG). Nerve conduction studies (NCS) can give a clue, such as low-amplitude compound muscle action potential (CMAP), and repetitive CMAPs after a single supramaximal stimulation. Needle electromyography (EMG) can show morphological variability (“ *jiggle* ”) of the motor unit action potential (MUAP). This mini-review aims to outline these electrophysiological tests. The abnormalities described are those used for the most common NMJ disorders. A brief clinical summary of the most common NMJ disorders is reported.

## NEUROMUSCULAR JUNCTION

The NMJ is a complex synapse between the terminal motor nerve and a muscle fiber membrane. The transmission of action potentials (AP) from the nerve to the muscle requires acetylcholine (ACh). The ACh is stored in the synaptic vesicles (SV) at the motor axon's terminal. A quantum of ACh represents about 10,000 molecules contained in one single SV. 1 It is released spontaneously and randomly, generating a 0.5 mV miniature endplate potential (MEPP) insufficient for muscle fiber activation. The SVs are distributed in three compartments. The primary, for immediate ACh release, comprises about 1,000 vesicles. The secondary replaces the primary in 1 to 2 seconds, comprising about 10,000 vesicles. The large tertiary comprises about 300,000 vesicles and slowly replaces the secondary compartment.

A quanta of ACh represents the number of SV released concurrently from the nerve terminal depending on the APs from the motor axons. A 60 to 300 SV quanta generates a not all-or-none endplate potential (EPP). The muscle fiber action potentials (MFAP) are generated in an all-or-none manner in response to the EPP. 2 3

The arriving motor axon APs at the presynaptic junction open the voltage-gated calcium channels (VGCC), resulting in calcium influx. 4 The higher the amount of calcium, the more ACh is released through the interaction with SNARE (SNAp + REceptor) proteins, such as the SNAP-25 (synaptosomal associated protein of 25kDa). ACh in the synaptic cleft binds to the muscle membrane's nicotinic ACh receptor (AChR), mainly located as groups in the crests of the folds of the post-synaptic membrane. 4 5 The bond of two ACh molecules to the AChRs results in sodium entrance, local depolarization, and an EPP generation. The EPP rises to a 25 to 45 mV peak in about 1 ms, causing the opening of a second class of ion channels in the muscle fiber, the voltage-gated Na _V_ 1.4 channels, thus initiating an AP in the muscle fiber. 6

The quantal ACh release depends on two presynaptic factors: the VGCCs and the number of SVs available for immediate release. The quantal release of ACh is four to five times greater than required to generate an EPP, the so-called SF. 3 As a result, the triggering of a MFAP is preserved under extreme physiological conditions. Acetylcholinesterase (AChE) breaks down the ACh, and the choline is retaken at the presynaptic terminal. 7

The SVs are progressively depleted from the primary compartment after a low-frequency repetitive nerve stimulation (LFRNS). Due to the SF, the EPP remains above the threshold, ensuring the generation of MFAPs. After 1 to 2 seconds, ACh mobilizes from the secondary compartment to replace the primary one. After high-frequency repetitive nerve stimulation (HFRNS), a depletion of ACh from the presynaptic nerve terminal occurs. Due to the quantal mobilization from the secondary compartment and the calcium accumulation, the neuromuscular transmission (NT) is maintained. As the calcium is eliminated from the presynaptic terminal in 100 ms, an HFRNS releases more calcium than its depletion, maintaining the NT. The same occurs immediately after a 10-second maximal voluntary isometric effort (MVIE).

## REPETITIVE NERVE STIMULATION

**Figure 1:**
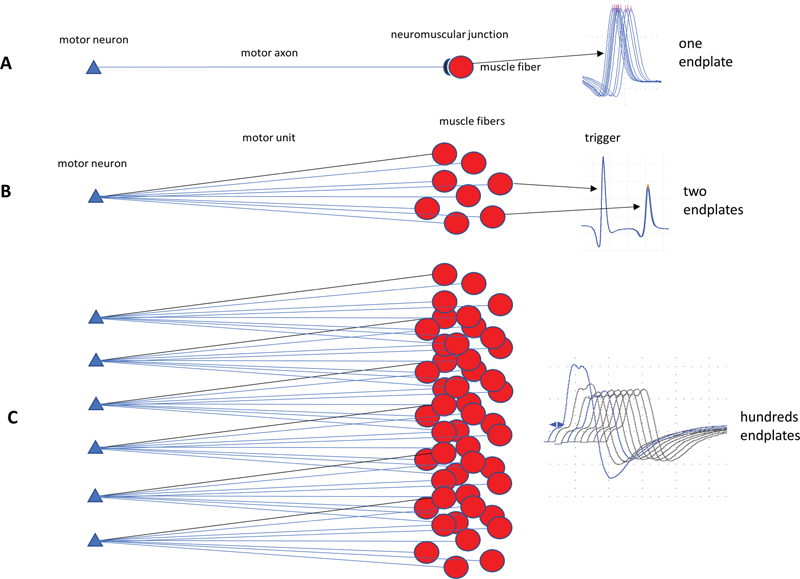
Basic concepts on neuromuscular junction electrophysiological evaluation. **(A)** . Single-fiber electromyography using the electrical activation technique analyses the time consecutive variation in just one muscle fiber (one endplate). **(B)** single-fiber electromyography using voluntary activation technique analyses the time consecutive variation in a pair of muscle fibers (two endplates). Techniques **(A)** and **(B)** can reveal abnormal jitter even in muscles without weakness. **(C)** Repetitive nerve stimulation analyses abnormal decrement after stimulation in a nerve trunk, so hundreds of muscle fibers from many motor units are tested. The decrement can be abnormal just in weak muscles.

The RNS is mandatory for disorders suspicious like myasthenia gravis (MG), Lambert-Eaton myasthenic syndrome (LEMS), congenital myasthenic syndromes (CMS), and botulism. Also, it should be done for patients with fixed bilateral and proximal weakness of uncertain etiology, for patients with MUAPs with short amplitude/duration suggestive of myopathy, 8 for patients with a reduced CMAP without neurogenic cause, and for patients with ptosis, diplopia or limb weakness from birth or childhood without a confirmed diagnosis. The RNS evaluates the impulse blocking in the NMJ from thousands of muscle fibers after supramaximal nerve trunk stimulation. Sensitivity is maximal when tested on weak muscles ( Figure 1 ).

Some technical factors are critical for a reliable RNS. The segment tested should be immobilized whenever possible, and the recording and stimulating electrodes must be well-fixed. The electrode wires should not touch the skin from the contracted muscles, avoiding spurious recordings. The patient should be instructed to avoid muscle contraction as the baseline variability leads to an inaccurate determination of the area or amplitude. A supramaximal stimulation must be certified. The temperature should be controlled at the recording site to ≥35°C, 9 as the cold limb reduces the AChE function. Anticholinesterase medications should be withheld for 12 hours before testing if this can be done safely. 9 10

**Figure 2:**
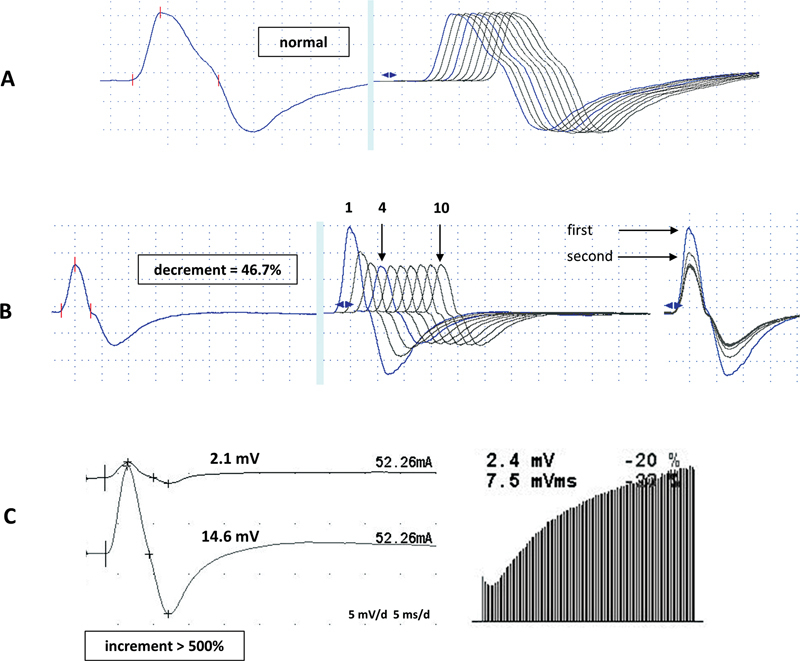
Repetitive nerve stimulation (RNS). **(A)** Normal low-frequency (2 Hz) RNS without increment or decrement. **(B)** Abnormal low-frequency (2 Hz) RNS showing a 46.7% decrement response from the fourth compared to the first response in a myasthenia gravis patient. Note that the most significant decrement occurred between 2 to 1 response (arrows), and the final percentage decrement is between 4 to 1 response (arrows). Observe the amplitude does not return to normal from 10 to 1 response. **(C)** In a Lambert-Eaton myasthenic syndrome case, small compound muscle action potential increases more than 500% after a 10-second maximal isometric voluntary effort (median nerve to *Abductor Pollicis Brevis* muscle) at the left bottom. Observe an initial decrement followed by a remarkable increment after a high-requency (20 Hz) RNS at the right bottom.

**Figure 3:**
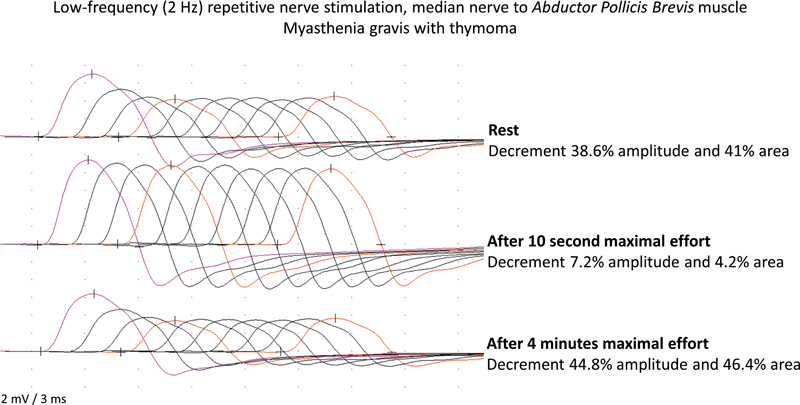
Abnormal decrement in a myasthenia gravis case by low-frequency repetitive nerve stimulation (2 Hz), median nerve to the *Abductor Pollicis Brevis* muscle. **First trace:** 38.6% amplitude decrement comparing the fourth to the first response. **Second trace:** there was a remarkable recovery immediately after a 10-second maximal voluntary isometric effort, showing just a 7.2% decrement comparing the fourth to the first response. **Third trace:** See the 44.8% amplitude decrement comparing the fourth to the first response after 4 minutes from the maximal voluntary isometric effort.

LFRNS (2 to 3 Hz) is performed to evaluate the NT function in post-synaptic NMJ disorders, like MG and most of the CMS ( Table 1 ). In each supramaximal stimulus, the ACh released will not be sufficient to elicit an EPP due to the reduced number of functioning AChRs. The LFRNS should be started in distal muscles in patients with generalized weakness. If normal, the examiner moved to the proximal muscles as the spinal-accessory nerve to *Trapezius* , the radial to *Anconeus* , and the axillary to *Deltoideus* . The most accessible proximal study is for the *Trapezius* muscle once the spinal-accessory nerve is superficial, and a supramaximal stimulation can be reached with less discomfort. The next group is for the facial muscles, including the *Nasalis* , the *Orbicularis Oculi* (OOc), or the *Orbicularis Oris* . The main issues for those muscles are the small CMAP amplitude, the lack of effective immobilization, and the need for the patient's collaboration, avoiding blinking, pulling a face, or making mouth movements. In cases with predominantly ocular or bulbar involvement and ocular MG (OMG) suspicion, the proximal and facial segments should be the first to be tested. The recommended frequency for the LFRNS is 2 to 5 Hz. 9 It is low enough to prevent calcium accumulation and sufficiently high to deplete the primary compartment. The train of stimuli could be 5 to 10. The train of ten had the advantage of comparing the 10th to the first CMAP amplitude ( Figure 2 ). If an abnormal decrement is found, the 10th amplitude never achieves the amplitude of the first response. Abnormality is considered when a reproducible 10% decrement in amplitude occurs, comparing the first CMAP amplitude to the fourth or fifth. 9 Notably, most decrement occurs between the first and the second CMAP. When the ACh mobilization from the secondary compartment begins to resupply the primary, the decrement initiates recovery, resulting in the characteristic “ *U-shaped* ” ( Figure 2 ). A reproducible decrement of less than 10% could be very suspicious 11 when the post-exercise response should be made after 1 minute of MVIE, followed by LFRNS every minute to the fourth or fifth. Typically, there is a decrement improvement in the first minute and a worsening by the third or fourth minute. 9 12 The reliability of an abnormal decrement could be confirmed by repeating the test after a 10-second MVIE as the decrement is repaired ( Figure 3 ).

**Table 1:** RNS findings in some primary and secondary neurotransmission dysfunction.

Frequency	Train	Use	Response	Sensitivity	Segment
2-5 Hz	5-10	GMG	Decrement “ *U-shaped* ”	90%	Distal/proximal
2-5 Hz	5-10	OMG	Decrement “ *U-shaped* ”	0 to 25%	Facial/proximal
2-5 Hz	5-10	Bulbar MG	Decrement “ *U-shaped* ” ***	80%	Proximal/facial
2-5 Hz	5-10	Most CMS	Decrement “ *U-shaped* ” or not	90.5%	Proximal/facial
2-5 Hz	5-10	LEMS	Decrement “ *U-shaped* ” or not	100%	Distal
20 Hz	100-200	LEMS	Increment >100%	100% Antibody (+)	Distal
20 Hz	100-200	LEMS	Increment 60 to 100%	100% Antibody (-)	Distal
20-50 Hz	100-200	Botulism	Increment 30 to 60%	depends on CMAP-A	Any
Single	1	LEMS	CMAP-A >100% *	85 - 95%	Distal
Single	1	Botulism	CMAP-A >30-60% *	depends on CMAP-A	Distal
Single	1	CMS - SCS	Repetitive CMAP **	25 to 50%	Distal
2-5 Hz	5-10	Denervation (ALS)	Decrement “ *U-shaped* ”	−	APB/ADM

Abbreviations: MuSK, muscle-specific tyrosine kinase; GMG, generalized myasthenia gravis; OMG, ocular myasthenia gravis; MG, myasthenia gravis; CMS, congenital myasthenic syndrome; SCS, slow-channel CMS; LEMS, Lambert-Eaton myasthenic syndrome; CMAP, compound muscle action potential; CMAP-A, CMAP amplitude; NCS, nerve conduction study; ALS, amyotrophic lateral sclerosis; APB, *Abductor Pollicis Brevis* ; ADM, *Abductor Digiti Minimi* .

Notes: *After 10-second effort; **After a single stimulus; ***Could be without “ *U-shaped* ” in cases of MuSK-MG.

The frequency for the HFRNS for a presynaptic NMJ disorder suspicion is usually 20 Hz, sometimes 50 Hz for botulism, but it is not recommended for an outpatient test. 9 Instead, a single supramaximal stimulation on the median nerve to the *Abductor Pollicis Brevis* muscle (APB) or the ulnar nerve to the *Abductor Digiti Minimi* muscle (ADM) at rest and after 10 seconds MVIE can demonstrate a CMAP increment ≥100% in LEMS ( Figure 2 ). The HFRNS can be considered in patients who are extremely weak to perform MVIE or are in a coma. Although a pre-synaptic disorder, LEMS presents a decrement response like MG in the LFRNS, sometimes lacking the typical “ *U-shaped* ”. 13 Some MuSKAb patients could also present an LFRNS without the “ *U-shaped* ”. 14

Other disorders may present decrement in the LFRNS ( Table 1 ), especially those with severe denervation, such as motor neuron disease. Plenty of new, immature, and unstable NMJs may reduce the SF. In all cases of limb-girdle weakness, an LFRNS should be done in one distal segment, as a fixed weakness may reveal MG. 15 Inflammatory myopathies, some myopathies (e.g., McArdle disease), some channelopathies (e.g., paramyotonia congenita and hyperkalemic periodic paralysis), some drugs (d-penicillamine, aminoglycosides, high dosage magnesium, 16 and nondepolarizing neuromuscular blocking agents), botulism and organophosphate poisoning can present with an abnormal to the slow- or HFRNS. In botulism, however, the abnormalities could be less pronounced, and in some cases, the tests cannot be done due to the absence of the CMAP.

## SINGLE-FIBER ELECTROMYOGRAPHY

SFEMG is a selective recording technique identifying single-fiber action potentials (SFAPs) 17 18 for calculating the jitter, the most sensitive parameter detecting an NT dysfunction, 18 as it tests single or a pair of endplates ( Figure 1 ). The single-fiber needle (SFE), with a small 25 µm diameter, a recording area of 0.0005 mm ^2^ , and a functional field of approximately 300 µm, records SFAPs. It is a biphasic spike with a rise time of 75 to 200 µs and a duration of approximately 1 ms. The closer the electrode is to the muscle fiber, the more selective the test is. Moving the needle as close to the muscle fiber assures a SFAP record (higher amplitude), not a summated from 2 or more. 11 18 The reference values were defined for various muscles, and the jitter values increased with age. 19

Due to virus or prion infection concerns, the reusable SFE was no longer allowed and was replaced by a disposable concentric needle electrode (CNE). The smallest recording area available (0.19 to 0.20 mm ^2^ ) is much larger and less selective in recording SFAPs. It is minimized by setting the filters 1,000 Hz to 10 KHz to exclude distant MFAPs. The jitter parameters required new reference values and more experienced practitioners, as the SFAPs are frequently summated and appropriately called “ *apparent* ” single-fiber action potentials (aSFAP). 20 Unlike the SFE, the CNE jitter does not increase with age. Since the first reports with CNE for measuring jitter, it became clear that there was less jitter. 20 21 22 23 24 25 26 27 28 29 30 31 Although inaccurate, SFEMG will be used here as synonymous with jitter analysis with CNE.

**Figure 4:**
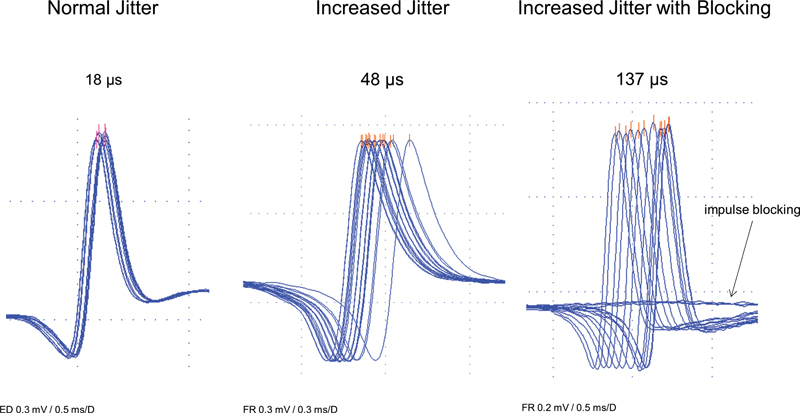
Abbreviations: ED, Extensor Digitorum muscle; FR, Frontalis muscle. Electrical activation technique showing a normal jitter, an increased jitter, and an increased jitter with impulse blocking in a case of myasthenia gravis. Observe the well-defined spikes with a parallel rising slope (depolarizing ascendent lines) and a constant shape at consecutive discharges.

When a single muscle fiber is electrically stimulated consecutively at 10 Hz, a slight variability in the latency of each response occurs due to changes in the amplitude of the EPP. This time variation, the neuromuscular jitter, has less than 5% contribution from the peripheral nerve or muscle fiber ( Figure 4 ).

The jitter is measured by a voluntary-activation technique (VAT), when 50 to 100 pairs of aSFAPs (two endplates) from the same motor unit are sequentially obtained by a slight muscle contraction. The mean consecutive difference (MCD) from those pairs represents the jitter. The electrical activation technique (EAT) can also measure the jitter, where the MCD of 50 to 100 sequential aSFAP latencies define it (one endplate).

The muscles usually studied are the ED, OOc, and *Frontalis* (FRO). If the SF is reduced, some EPPs will not reach the threshold and fail to depolarize muscle fibers (impulse blocking). The impulse blocking results in muscle weakness. The VAT is the first choice for measuring jitter due to a few examiner's pitfalls. The examiner triggers one aSFAP and, by gently moving the needle, tries to find another aSFAP time-locked to the first from some motor unit. Due to the time variation of the two endplates, the interpotential interval (IPI) variability is the jitter. The jitter is abnormal when the mean jitter of the 20 pairs is above the reference limit. The jitter is also abnormal if more than 10% of the 20 individual pairs exceed the reference limit. The mean and individual jitter reference values are already published in a multicenter study. 22 CNE sometimes records multiple aSFAPs; the one with the most abnormal jitter should not be used as the triggered spike.

The EAT is done by stimulating motor axon twigs intramuscularly using a monopolar needle electrode, especially for large muscles. Rectangular pulses deliver the stimulation of 0.05 to 0.10 ms at 10 Hz, usually at an intensity of 1 to 3 mA. The recording CNE is inserted in the twitching area around the stimulation electrode. For the tiny facial muscles, the motor axons are stimulated percutaneously by a bar electrode on either the zygomatic (OOc muscle) or the temporalis (FRO muscle) facial nerve branches. The stimulation is delivered with rectangular pulses of 0.10 ms duration until a twitching can be seen in the palpebrae, commonly at an intensity of 5 to 7 mA. For the OOc muscle, the recording CNE is inserted about 2 to 3 cm surrounding the eyeball, starting in the lateral part. For the FRO muscle, the recording CNE is inserted in a defined sequence in any part of the forehead. Most of the studies on EAT were done by Jože Trontelj. 32 33 As only one aSFAP is studied each time, the stimulus-to-response latency is measured rather than the IPI. The number of aSFAPs studied is 30. The test is abnormal if the mean jitter of the 30 recordings exceeds the reference limit or if more than 10% of the 30 individual jitter values exceed the reference limit (22). Recording many aSFAPs (multi-spikes) with a distinct threshold for each one is expected in EAT. The examiner must ensure a supramaximal stimulation in any of those spikes and acquire one each time. The mean jitter value in the EAT is less than the VAT. The jitter by the VAT is theoretically increased by a root-squared of 2 or 1.41 times. So, for a mean EAT jitter of 20 µs, the expected VAT jitter is 28.2 µs. The EAT is appropriate for patients with difficulty maintaining mild voluntary contraction, patients with movement disorders, consciousness disorder patients, and children, or when the examiner needs the jitter evaluation at various discharge frequencies. This technique has more pitfalls and should be done by doctors with considerable experience. 34

As the aSFAP frequently represents summated spikes, new criteria for accepting the spikes were established when recording using a CNE. 17 35 The rise should be less than 300 µs. They should be well-defined, have a parallel depolarizing ascendent line, and maintain a constant shape at consecutive discharges. The aSFAPs with notches, shoulders, or a double peak must be excluded as it represents a summation ( Figure 4 ). The time variation of the aSFAP calculation (jitter) could be done by the voltage level technique (usually called “level”) or multipeak detection technique (usually called “peak”). Pitfalls occur in both activation techniques; some are specific to the EAT or VAT. 17 34 35 The skin temperature should be kept above 32°C. The EAT firing rate for post-synaptic (MG and most CMS) suspicion is 10 Hz. The patient must be asked about botulinum neurotoxin (BoNT) use. It is not mandatory, 36 but the patient should interrupt the AChE inhibitors 12 hours before the test.

The flip-flop and the triangular recordings are relatively common in VAT and should not be used for jitter calculation. The interdischarge interval (IDI) should be constant and ideally between 5 to 10 Hz. The IPI must be less than 4 ms to avoid interference with the velocity recovery function (VRF). The correlation between the IPI and IDI interval can be seen during the exam and should show a straight line without trends. The acceptable aSFAPs should follow the rise time, amplitude, and morphology criteria. Impulse blocking does not occur with jitter less than 80 µs, mainly representing some technical pitfall. The maximal jitter value should be 150 µs for all values above it. It will prevent the VRF effect from increasing the jitter values artificially due to impulse blocking. The mean sorted difference (MSD) representing the aSFAPs according to the mean IDI order should be chosen when it is less than the MCD. The jitter values of less than five µs must be discarded as they represent a recording in the same muscle fiber due to splitting. A common and critical pitfall in the EAT is a submaximal activation leading to a false increased jitter with impulse blocking. The f-waves are usually present in 5% of the recordings and could interfere with the jitter measuring. The “ *axon reflex,* ” seen in intramuscular stimulation, can be challenging for measuring jitter in an inexperienced examiner. Also, a common pitfall occurs when the tip of the CNE slightly moves away from the muscle fiber and returns to the previous position, causing points with the VRF effect (increase and decrease latencies). Intramuscular stimulation can directly activate muscle fiber, and accordingly, with no jitter; all supposed jitter ≤5 µs must be discarded.

Some conditions may interfere with the jitter measurement:

- The jitter is abnormal in muscles with active denervation, mostly less than 88 µs. 37 In muscles with chronic reinnervation, jitter is abnormal in 75%, mostly less than 65 µs. 37
- In muscles directly injected with the BoNT, the jitter is abnormal in all cases, usually with a high percentage of impulse blocking in the first weeks. The injected muscle can never be used for measuring jitter. Despite the BoNT loss effects in months, some motor unit reorganization can occur. 38 A jitter was found abnormal in 40% of a neighboring muscle from a BoNT injection, as high as 43.7 µs (maximum reference, 28 µs). The muscle can be used for measuring jitter after 11 months from the last injection, and the reference limit is raised by 33%.38 A jitter was abnormal in 14% of a distant muscle from a BoNT injection, as high as 41.4 µs (maximum reference, 30 µs). The muscle can be used for measuring jitter after eight months from the last injection, and the reference limit is raised by 10%. 38
- Jitter cannot be used as an electrodiagnostic test in muscles presenting MUAPs suggestive of myopathy.
- Check all the patient's continuous drugs that could interfere with the NT, e.g., calcium channel blockers (verapamil and amlodipine) or AChE inhibitors. 39 ( Table 2 )

**Table 2:** Jitter findings in some primary and secondary neurotransmission dysfunction.

EAT	VAT	Use	Response	Sensitivity	Segment
10 Hz	Regular	GMG	Increased jitter and blocking	99-100% (2 muscles)	ED, FR or OOc
10 Hz	Regular	OMG	Increased jitter and blocking	100% (2 muscles)	FR and OOc
10 Hz	Regular	Bulbar MG	Increased jitter and blocking	95%	FR and OOc *
10 Hz	Regular	CMS	Maximal obtained 98.5 µs	95.2%	FR and OOc
2 Hz	Regular	LEMS	Increased jitter and blocking	99 to 100%	ED
10 Hz	−	Active Denervation	Maximal obtained 88 µs	100%	Given muscle
10 Hz	−	Chronic Reinnervation	Maximal obtained 65 µs	75%	Given muscle
10 Hz	Regular	Myopathy	Increased	Never used	−
−	Regular	CPEO	Maximal obtained 46.9 µs	25%	OOc
10 Hz	Regular	BoNT injected muscle	Abnormal jitter in all (weeks)	Never used	−
−	Regular	BoNT near muscle	Increase 33% to the ULN *	−	−
−	Regular	BoNT distant muscle	Increase 10% to the ULN **	−	−
−	Regular	Verapamil/Amlodipine	Borderline jitter 50 µs (SFE)	6.25 to 12.50%	ED

Abbreviations: EAT, electric activation technique; VAT, voluntary activation technique; MG, myasthenia gravis; GMG, generalized MG; ED, *Extensor Digitorum* muscle; FR, *Frontalis* muscle; OOc, *Orbicularis Oculi* muscle; OMG, ocular MG; CMS, congenital myasthenic syndrome; LEMS, Lambert-Eaton myasthenic syndrome; CPEO, chronic progressive external ophthalmoplegia; BoNT, botulinum neurotoxin; ULN, upper limit of normality; SFE, single-fiber electrode.

Notes: *After 11 months from the last injection; **After 8 months fro the last injection.

## NEUROMUSCULAR JUNCTION DISORDERS

NMJ disorders are rare. They are related to the immune system, such as MG and LEMS; genetic, such as CMS; toxins, such as botulism and some snake venoms 40 ; and drugs, such as d-penicillamine, calcium-blockers, and some antibiotics. The main electrophysiological findings in the four leading causes of NT dysfunction are shown in Table 3 .

**Table 3:** Main electrophysiological findings in the four most common neuromuscular junction disorders.

Disease	CMAP	LFRNS	HFRNS	SFEMG	FP/PSW	MUAP
MG	Normal	Decrement	Improve	Abnormal	No	Some myopathic
LEMS	Reduced	Decrement	Increment	Abnormal	No	Some myopathic
Botulism	Reduced	Decrement	Increment	Abnormal	Yes	Some myopathic
CMS	Rep CMAP *	Decrement **	Improve	Abnormal	No	Some myopathic

Abbreviations: CMAP, compound muscle action potential; Rep, repetitive; FP, fibrillation potentials; PSW, positive sharp wave; MUAP, motor unit action potential; MG, myasthenia gravis; LEMS, Lambert-Eaton myasthenic syndrome; CMS, congenital myasthenic syndromes; LFRNS, low-frequency repetitive stimulation; HFRNS, high-frequency repetitive stimulation.

Notes: * Slow-channel CMS; ** Most CMS.

### Myasthenia gravis

MG is an autoimmune disease caused mainly by a direct attack of immunoglobulin G against the AChRs, generally through complement activation. Approximately 85 to 92% of all MG patients are positive for AChR antibodies (AChR-Ab). In these cases, about 10% present concomitant thymoma. About 50% of AChR-Ab negative patients exhibit muscle-specific tyrosine kinase (MuSK) antibodies, a protein involved in AChR clustering. A third and fourth antibody against low-density lipoprotein receptor-related protein 4 (LRP-4) and against agrin can be detected. In double seronegative for the first two, 15% was positive for either LRP4 or agrin antibodies, and 13% had both positive. 41

The AChR-Ab positive patients present fluctuating weakness of the extraocular, bulbar, and proximal limb muscles. Ptosis and diplopia are the first symptoms in more than 50%, and 90% of cases will present them throughout the disease. The high incidence of symptoms in the ocular muscles is an unsolved question; a distinctive finding in some extraocular muscle fibers is the presence of both fetal- and adult-type AChR. 4 Approximately 15% of MG patients remain as OMG. Bulbar weakness is the second most common symptom, with difficulty swallowing, chewing, and speaking. If an OMG is stable for two years, probably do not go to GMG. Limb weakness is symmetrical and proximal. The MuSKAb-positive patients are predominantly Afro-American young women with a more severe clinical picture. Most have oculobulbar weakness with atrophy of the face and tongue. Severe neck, shoulder, and respiratory muscle weakness are more frequent than AChR-Ab cases. Patients are often unresponsive or even intolerant to AChE inhibitors, and some even worsen. 42 43 Most LRP4- and agrin-antibody-positive patients (89%) developed GMG. After a standard MG treatment, 82% improved to MGFA class I or II during a mean follow-up of 11 years. 41

Jitter measurement is the most sensitive test NT function in MG, 18 44 45 being abnormal in up to 99% of cases. 17 18 35 46 For patients without abnormal decrement and negative antibodies, the increased jitter will support the MG diagnosis in cases responsive to prednisone. Despite the localized weakness, as in OMG, Weinberg et al. 47 found a 70% increased jitter in the ED muscle, emphasizing abnormal jitter in non-symptomatic muscles. Padua et al. 48 found 100% abnormal jitter in the OOc muscle for the OMG cases. Lo et al. 49 found 100% abnormal jitter in the OOc muscle for the bulbar MG cases. In cases of OMG, abnormal jitter was more frequent in the OOc than in the FRO muscle. 31 The jitter was reduced by 15.4 µs after MG therapy with mycophenolate mofetil, indicating a robust clinical correlation. 50 Abnormal jitter was found in 93% and 99% of OMG and GMG patients, respectively, when the ED and OOc muscles were analyzed. 51 For MuSK patients, jitter measurement should be done with facial muscles, sometimes on both sides. Jitter was abnormal in 75% of the OOc muscle and 8% of the ED muscle for MuSK patients. 52 For MuSK patients, the LFRNS should be made on facial muscles. 53 In a high suspicion of MG with a seronegative AChR-Ab, the jitter measurement should be done in the facial muscles. 52 54 55

The sensitivity of a given electrophysiological test in 97 MG cases, 46 either OMG or GMG, is shown in Table 4 . The sensitivity of the LFRNS increases by careful muscle selection depending on the most involved territory, ocular, bulbar, or generalized. It also increases by about 7%, repeating the test four minutes after MVIE. 12 Some suggested a lower cutoff value (7 or 8%) for the facial muscles. 56 However, the low CMAP amplitude in these muscles prevents a high-quality record. The percentage of LFRNS decrement did not significantly correlate with AChR-Ab titers or clinical symptom severity. 57 For MG patients with LRP-4 antibodies, one study found that LFRNS was normal in all 17 cases.

**Table 4:** Abnormality in each electrophysiological test in 97 myasthenia gravis cases. 46

Abnormality	MG (97)	GMG (85)	OMG (12)
Decrement	78.4%	85.9%	25%
Any jitter parameter	93.8%	92.9%	100%
AChRAb or MuSKAb	86.1%	91.8%	50%

Abbreviations: MG, myasthenia gravis; GMG, generalized MG; OMG, ocular MG; AChRAb, acetylcholine anti-receptor antibody; MuSKAb, muscle-specific tyrosine kinase antibody.

### Lambert-Eaton myasthenia syndrome

LEMS is an autoimmune NMJ disorder, mostly presenting IgG antibodies against VGCC type P/Q- and N-, interfering with the presynaptic quantal release of calcium-dependent ACh, resulting in a reduced EPP in the postsynaptic membrane. LEMS presented a fixed proximal weakness in the lower limbs; the deep reflexes are reduced or absent; autonomic symptoms such as dry mouth are common. Ptosis, dysarthria, and dysphagia are uncommon. After exercise, muscle strength and deep reflexes can transiently improve. 13 LEMS usually affects adults over 40 (70% of men). SCLC is found in about half of patients. SCLC expresses antibodies against VGCC type P/Q- and N- causing the NMJ and autonomic symptoms. Patients without cancer are usually younger women with primary autoimmune diseases. Antibodies against VGCC P/Q-type are found in 85% of cases with LEMS, with or without SCLC.

The CMAP obtained after any nerve stimulation has a small amplitude due to the reduction of the quantal release of ACh. It is easily seen in the median nerve to APB muscle in 95% and ulnar nerve to ADM muscle in 85% of cases 58 as a low CMAP amplitude in routine NCS 18 44 59 in 85 to 95% of cases. After a 10-second MVIE, another stimulus causes a CMAP amplitude increment usually greater than 100% ( Figure 2 ). A decrement response is found after LFRNS, sometimes without the typical “ *U-shaped* .” These electrophysiological abnormalities are found in 98% studying either APB or ADM muscles. 58 The calcium entrance is higher than the calcium output at a higher firing rate, either HFRNS or MVIE.

EMG has no spontaneous activity, and the MUAPs are sometimes unstable (“ *jiggle* ”) 60 and of small amplitude, short duration, and polyphasic, similar to myopathy. In 85% of LEMS patients, the increment was greater than 100%. 59 If the increment is limited to 60%, 97% of the LEMS patients reach this value. Considering that only 0.74% of the MG cases had an increment >60%, rare patients with an increment between 60 to 100% could be found in MG. All LEMS cases with positive P/Q-type VGCC antibodies had an increment of ≥100%. In contrast, all LEMS cases without P/Q-type VGCC antibodies had an increment ≥60%. In LEMS, jitter is abnormal, with frequent impulse blocking. SFEMG is barely necessary 18 as the electrodiagnosis can be made at ordinary NCS.

The jitter and the impulse blocking are improved at high-frequency (20 Hz) jitter stimulation. 59 61 62 63 An inverse situation, a low-frequency (1 to 2 Hz) jitter stimulation, causes an increased jitter with impulse blocking. 63 However, there are exceptions, and LEMS and MG cannot be fully differentiated by the jitter stimulation frequency. 61

### Botulism

Botulism is a disorder of NT characterized by afebrile, symmetric descending weakness, respiratory failure, and autonomic dysfunction. 64 Botulism is caused by a BoNT from *Clostridium Botulinum* , which prevents the presynaptic release of ACh from both somatic and autonomic synapses, blocking the molecular chain for it to happen. The BoNT-contaminated food intake is one of the causes. It can also stem from infected wounds and intravenous illicit drugs. In some countries, a common form of botulism is infantile, when spores are introduced into the gastrointestinal tract, where they germinate and produce the BoNT that is absorbed. Spores are widespread in the soil and are often found in honey. In adult botulism, symptoms appear two to seventy-two hours after BoNT ingestion or its production in wounds. Nausea, vomiting, and abdominal pain are common initial symptoms, progressing to blurred vision, diplopia, and dysarthria. Rapid progression to descending weakness occurs, with flaccid areflexic tetraparesis, respiratory involvement, and ophthalmoplegia. The pupils become paralyzed in 50% of cases. Other manifestations of parasympathetic dysfunction are paralytic ileus, reduced salivation, and difficulty in visual accommodation. The disease progresses in one to two weeks, with slow recovery in many months. MG is the most relevant differential diagnosis but barely presents with an acute presentation, and there are no autonomic symptoms. Infantile botulism rarely presents with severe findings similar to food or wound botulism. The most frequent symptoms are hypotonia, reduced movement, weak crying, and constipation.

Its electrophysiological evaluation is similar to LEMS, and decrement can also be seen in LFRNS. Increment occurs after a 10-second MVIE or HFRNS, mostly less than 60%. The typical electrophysiological findings usually appear in mild cases and at the initial phase. The EMG frequently shows fibrillation potentials and positive sharp waves due to chemical denervation. MUAPs may be normal or have a myopathy-like pattern like other NMJ disorders. The CMAP is usually absent for severe suspected, preventing the motor NCS evaluation. The jitter obtained by electrical activation is most helpful in detecting a dysfunction in NT 65 66 and reveals increased jitter with impulse blocking.

### Congenital myasthenic syndromes

**Figure 5:**
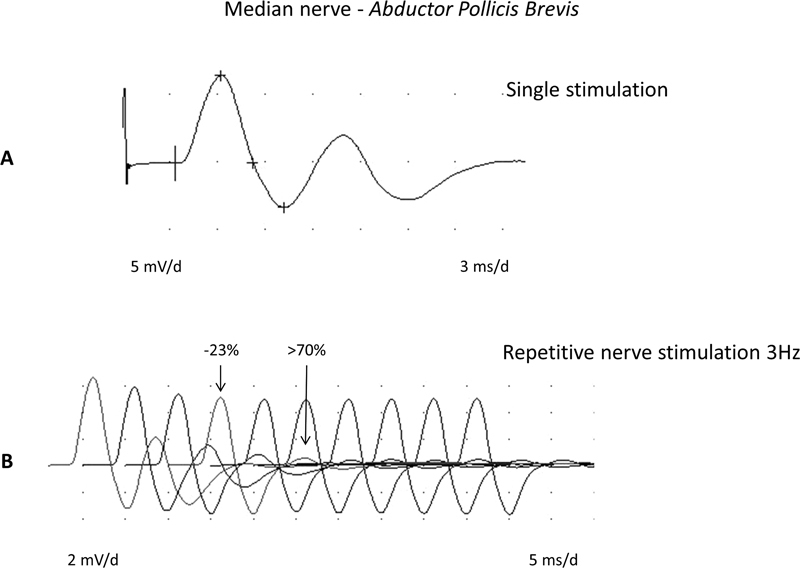
Congenital myasthenic syndrome (slow-channel syndrome) showing a typical repetitive compound muscle fiber action potential on a single supramaximal median nerve stimulation (recording, *Abductor Pollicis Brevis* ). The low-frequency (3 Hz) repetitive nerve stimulation reveals a 23% decrease when the fourth response is compared to the first. Observe that the second response had an even worst decrement.

CMS comprehends a group of NMJ disorders caused by monogenetic defects that lead to reduced SF in NT. They are suspected in cases with weakness fluctuation or previously suspected MG but lacking antibodies and response to immunotherapy. The age of presentation before two years old should also raise suspicion. The main clinical features shared by most CMS are fatigable muscle weakness and muscle hypotrophy. Proximal, ocular, and bulbar muscles are frequently affected, but only ocular presentation is not expected. 67 Among 35 genes associated with CMS, *CHRNE* is the most frequently related, followed by *RAPSN* , *DOK7* , *COLQ* , and *GFPT1* . 68 In Brazil, AChR deficiency due to *CHRNE* defects is the most prevalent, with variant c.130dupG presenting in up to 70% of cases. 69 70 AChR deficiency related to *CHRNE* is an early-onset syndrome with ptosis, ophthalmoplegia, bulbar, and limb weakness, usually mild to moderate severity. 70 Defects on *RAPSN* reduce the number and density of AChRs in the postsynaptic membrane folds. One phenotype is early-onset neonatal hypotonia, which can present respiratory failure at birth, episodic apnea, or arthrogryposis multiplex congenita. Another phenotype is limb weakness, sometimes with ocular symptoms, in teenagers or adults. 71 The *DOK7* CMS patients have significantly flattened postsynaptic membrane with reduced folds and clefts. The typical phenotype is early or late-onset limb-girdle weakness and ptosis, sparing ocular and facial muscles. 67 AChE deficiency due to *COLQ* defects presents diffuse muscle weakness, with severe involvement of axial and ocular muscles; in some cases, there is a slow pupillary light response. 68 Glycosylation deficiency can affect the NMJ, and the main gene-related is *GFPT1* . Usually, a limb-girdle weakness without ptosis and ocular involvement is found; muscle biopsies can reveal tubular aggregates. 68 Defects in the AChR subunits could result in the slow-channel (SCCMS) or fast-channel CMS (FCCMS) due to the abnormal opening time of these receptors. From a clinical point of view, FCCMS is very similar to AChR deficiency. On the other hand, early or late onset of severe neck, wrist, and finger extensor weakness is typical in SCCMS, sometimes with progressive ventilatory insufficiency. 68 Most CMS have a postsynaptic dysfunction. Caldas et al. 72 found an abnormal decrement after LFRNS in 90.5%, studying the *Deltoideus* , *Trapezius* , OOc, and *Nasalis* muscles, and an abnormal jitter in 95.2% studying the VAT OOc muscle. Patients with AChE deficiency or SCCMS may present repetitive CMAP after a single supramaximal stimulus in routine NCS ( Figure 5 ) and an abnormal jitter in most cases.

Most CMS patients improve with pyridostigmine, despite some not or even worse, notably SCCMS, *COLQ* -CMS, and *DOK7* -CMS. Besides AChE inhibitors, 3,4-diaminopyridine can be used. This drug must be avoided on FCCMS and those not responsive to pyridostigmine. Ephedrine and salbutamol are effective in several CMS, particularly in endplate AChE deficiency and in patients with *DOK7* -CMS.
